# The altered gut microbiota of high-purine-induced hyperuricemia rats and its correlation with hyperuricemia

**DOI:** 10.7717/peerj.8664

**Published:** 2020-03-06

**Authors:** Xiu Liu, Qiulan Lv, Hongyan Ren, Liu Gao, Peng Zhao, Xiaomin Yang, Guanpin Yang, Daxing Xu, Guangtao Wang, Wan Yang, Pengjun Wang, Zenglan Wang, Shichao Xing

**Affiliations:** 1 Department of Pathogenic Biology, School of Basic Medicine, Qingdao University, Qingdao, China; 2 Affiliated Hospital of Qingdao University, Qingdao, China; 3 Shanghai Itechgene Technology Co.,Ltd, Shanghai, China; 4 Medical Department of 3SBio Group, Shanghai, China; 5 The Key Laboratory of Mariculture of Ministry of Education, Ocean University of China, Qingdao, China; 6 Institute of Sports Medicine and Health, Qingdao, China; 7 School of Cardiovascular Medicine and Science, King’s College London, London, United Kingdom

**Keywords:** Gut microbiota, Hyperuricemia, Correlation, Change, Gut microbiota disorder, Fecal microbiota transplantation

## Abstract

Some studies on the hyperuricemia (HUA) have focused on intestinal bacteria. To better understand the correlation between gut microbiota and HUA, we established a HUA rat model with high-purine diet, and used 16S rRNA genes sequencing to analyze gut microbiota changes in HUA rats. To analyze the potential role played by gut microbiota in HUA, we altered the gut microbiota of HUA rats with antibiotics, and compared the degree of uric acid elevation between HUA and antibiotic-fed HUA rats (Ab+HUA). Finally, we established a recipient rat model, in which we transplanted fecal microbiota of HUA and normal rats into recipient rats. Three weeks later, we compared the uric acid content of recipient rats. As a result, the diversity and abundance of the gut microbiota had changed in HUA rats. The Ab-fed HUA rats had significantly lower uric acid content compared to the HUA rats, and gut microbiota from HUA rats increased uric acid content of recipient rats. The genera *Vallitalea*, *Christensenella* and *Insolitispirillum* may associate with HUA. Our findings highlight the association between gut microbiota and HUA, and the potential role played by gut microbiota in HUA. We hope that this finding will promote the isolation and culture of HUA-related bacteria and orient HUA-related studies from being correlational to mechanistic. These steps will therefore make it possible for us to treat HUA using gut microbiota as the target.

## Introduction

Hyperuricemia (HUA) is a metabolic disease that results due to purine metabolism disturbances, characterized by an increase in uric acid synthesis and a decrease in the excretion of uric acid (De Oliveira & Burini, 2012). The disease can easily develop into gout, which can cause acute gouty arthritis, joint deformity, uric-acid nephropathy and other serious manifestations (Roubenoff et al., 1991; Li et al., 2015). Therefore, HUA is a serious threat to human health. With the changes in lifestyle and diet structure and the prevalence of alcoholism, obesity and hypertension, HUA has become epidemic (Miao et al., 2008). As documented previously, the prevalence of HUA in Italy is 11.9%, and in China it is as high as 13.3% (Kim, Kang & Kim, 2018). HUA has become one of major metabolic diseases following diabetes; however, its pathogenesis is still unclear.

Diverse microorganisms like bacteria, viruses and fungi inhabit the human intestinal tract. Since many of the gut microorganisms cannot be cultured, gut microbiota can instead be identified by 16s rDNA sequencing. A large number of studies on the 16S rDNA sequence have shown that the human intestinal tract is mainly composed of the phyla Bacteroidetes and Firmicutes, although there are trillions of microorganisms live in it (Torres-Fuentes et al., 2017). In addition to analyzing microbial composition, 16s rDNA sequencing can also be utilized to measure microbial diversity, which is a measure of the number, type and evenness of the gut microorganisms (Deering et al., 2019). Therefore, changes in intestinal microbiota in diseased individuals can be assessed by analyzing the microbial composition and diversity, and exploring the relationship between microorganisms and the host.

In recent years, increasing number of studies have reported the involvement of intestinal microorganisms in several metabolic diseases including diabetes, obesity and lipid metabolism disorder, highlighting a key role of microbiota in host metabolism (Torres-Fuentes et al., 2017). Because one third of uric acid is excreted through intestinal tract, HUA-related studied have focused on analysis of intestinal bacteria in recent years (Hosomi et al., 2012). Previous studies found that gout patients presented with dysregulation of intestinal microbiome, which in turn served as an indicator for gout. Similarly, a potential probiotic was found to improve fructose-induced HUA by reducing intestinal inosine (Shao et al., 2017; Guo et al., 2016; Wang et al., 2019). The findings from these early studies implied that the gut microbiota could be a new target for the treatment of HUA and gout. However, these early studies found no direct evidence showing a role of gut microbiota in HUA and gout.

This present study was performed with an aim to explore the changes in gut microbiome in HUA and find any correlation between the gut microbiota and HUA. We analyzed the gut microbiome of HUA rats, and then compared the degree of UA elevation between HUA and Ab-fed HUA rats. Finally, we observed the uric acid content of rats transplanted with the gut microbiota of HUA rats. Based on our findings, we suggest that the gut microbiota does play a role in HUA.

## Materials and Methods

### Animals

Animals for this study were obtained from Qingdao qinda biotechnology Co.,Ltd. All experimental animals were approved by the Animal Research Ethics Committee of the Affiliated Hospital of Qingdao University; Approval No. AHQU-MAL2017022). All animals were housed individually in a sterile and ventilated cage at standard temperature (24 ± 1  ^∘^C) and humidity (65%–70%) with lights on 12-h light-dark cycle. Before the experiment, the rats were fed normal forage and water. The rats that had been anesthetized with inhaled diethyl ether were sacrificed by decapitation after the experiment.

### Hyperuricemia rat modeling and feces and blood sampling

Forty-eight 6-week-old male Wistar rats were assigned to the hyperuricemia group (HUA, *n* = 29) and the normal group (N, *n* = 19). The HUA group was fed yeast-rich forage and gavaged with purine at 100 mg/(Kg ⋅ d), whereas the N group was fed normal forage and gavaged with the same volume of water. After 5 weeks, fresh feces were obtained by stimulating the anus. Rats that had been fasted overnight were anesthetized briefly with inhaled diethyl ether, and the blood samples were collected from the intra-orbital retrobulbar plexus. Blood was used for uric acid measurements while feces were stored at −80 °C and used for analysis of gut bacterial community.

### Antibiotics interference of gut microbiota and feces and blood collection

32 male Wistar rats (6 weeks old) were randomly divided into four groups, with 8 rats in each group. The normal group (N) was fed water, whereas the hyperuricemia group (HUA) was administrated yeast-rich forage and gavaged with purine at 100 mg/(Kg ⋅ d). Antibiotics were used as before (Hu et al., 2015), with minor modifications. Antibiotics-fed hyperuricemia group (Ab+HUA) was administrated yeast-rich forage, gavaged with purine at 100 mg/(Kg ⋅ d) and supplemented with water with antibiotic mixture (ampicillin 250 mg/kg + neomycin 250 mg/kg + metronidazole 50 mg/kg). Antibiotics-fed normal group (Ab+N) was gavaged with water supplemented with antibiotic mixture (ampicillin 250 mg/kg + neomycin 250 mg/kg + metronidazole 50 mg/kg). The experiment lasted five weeks during which purine and antibiotic mixture were administered once daily. Throughout the experiment, orbital blood and fresh feces were collected once a week and used to measure the biochemical indicators and analyze the intestinal microbiota, respectively.

### Recipient rat modeling and fecal microbiota transplantation

Germ-free intestinal environment of recipient rats was stimulated following reported methods with minor modifications (Zhao et al., 2018). Twenty normal rats were given water supplemented with antibiotic mixture (ampicillin 250 mg/kg + neomycin 250 mg/kg + metronidazole 50 mg/kg) daily by gavage. Twelve days later, the fecal microbiota of rats was analyzed to ensure that the intestinal environment was as sterile as possible. Then twenty recipient rats were randomly assigned to a normal microbiota transplantation group (NMT) and a hyperuricemia microbiota transplantation group (HMT), with each group containing 10 rats. Fresh feces from the HUA and normal rats in the previous experiment were collected separately. As reported previously, the fresh feces were immediately homogenized in pre-reduced PBS at 1 ml per pellet (Yano et al., 2015). Fecal solution (one mL) was gavaged to all the recipient rats for one consecutive week. NMT group was gavaged with fecal solution of N rats, whereas HMT group was gavaged with the fecal solution of HUA rats. Fresh feces and orbital blood were collected every other week after continuous transplantation for one week. All rats were fed normal forage and water throughout the experiment.

### 454 pyrosequencing

DNA from the frozen feces was extracted using the E.Z.N.A.^^®^^ Stool DNA Kit (OMEGA, America). The quality of DNA was analyzed using a NanoDrop-2000 spectrophotometer (Thermo Scientific). The V1-V3 variable regions of the 16S rRNA genes were amplified using the primers 27F (5′-AGA GTT TGA TCC TGG CTC AG -3′) and 533R (5′-TTA CCG CGG CTG CTG GCA C-3′) in a 20 µL mixture containing 4 µL of Phusion HF buffer, 0.8 µL of forward primer (5 µM), 0.8 µL of reverse primer (5 µM), 0.4 µL of dNTP (10 mmol/L each), 0.2 µL of Phusion DNA polymerase, and 10ng of fecal DNA as template. The reaction was performed with following PCR program: denaturation at 98 °C for 30 s, followed by 25 cycles of denaturation at 98 °C for 10 s; annealing at 55 °C for 30 s and extension at 72 °C for 30 s; and a final extension at 72 °C for 5 min. Agarose gel (2%) electrophoresis was used to purify the PCR product, and the purified product was sequenced on Roche GS-FLX platform.

### High-throughput sequencing on Illumina MiSeq platform

DNA was extracted with the E.Z.N.A.^^®^^ Stool DNA Kit. 16S rRNA genes (V3-V4 variable regions) were amplified with the primers 338F (5′-ACT CCT ACG GGA GGC AGC AG -3′) and 806R (5′-GGA CTA CHV GGG TWT CTA AT -3′). The amplification reaction was performed in a 25 µL volume containing 12.5 µL of Pusion Hot start flex 2X Master Mix, 2.5 µL of forward primer, 2.5 µL of reverse primer, and 50 ng of template DNA. The reaction was performed with following PCR program: denaturation at 98 °C for 30 s, followed by 35 cycles of denaturation at 98 °C for 10 s, annealing at 54 °C for 30 s and extension at 72 °C for 45 s, and a final extension at 72 °C for 10 min. The PCR product was purified through agarose gel (2%) electrophoresis and commercial sequencing was conducted on Illumina MiSeq platform.

### Sequence analysis

The raw sequencing reads were processed by splicing and filtering with the clean data clustered into operational taxonomic units (OTUs) by ≥97% sequence similarity. The gut microbial diversity was determined by the abundances of OTUs in different samples. Species annotation was performed by comparing the representative sequence of each OTU with the 16S rDNA database. LEfSe analysis identified the discrepant microbial species between groups. The Spearman’s rank correlation analysis was used to find the relationship between the gut microbiota and hyperuricemia.

### Statistical analysis

All data are presented as the mean ± standard deviation (SD). All biochemical data and microbial data from the two groups were compared using Student’s unpaired *t*-test and Wilcoxon rank-sum test, respectively. Similarly, biochemical data and microbial data from multiple groups were compared using one-way ANOVA and Kruskal-Wallis test, respectively. *P*-values <0.05 were considered statistically significance. The data was analyzed using SPSS and R software.

## Results

### The uric acid content of HUA rats increased significantly

**Figure 1:**
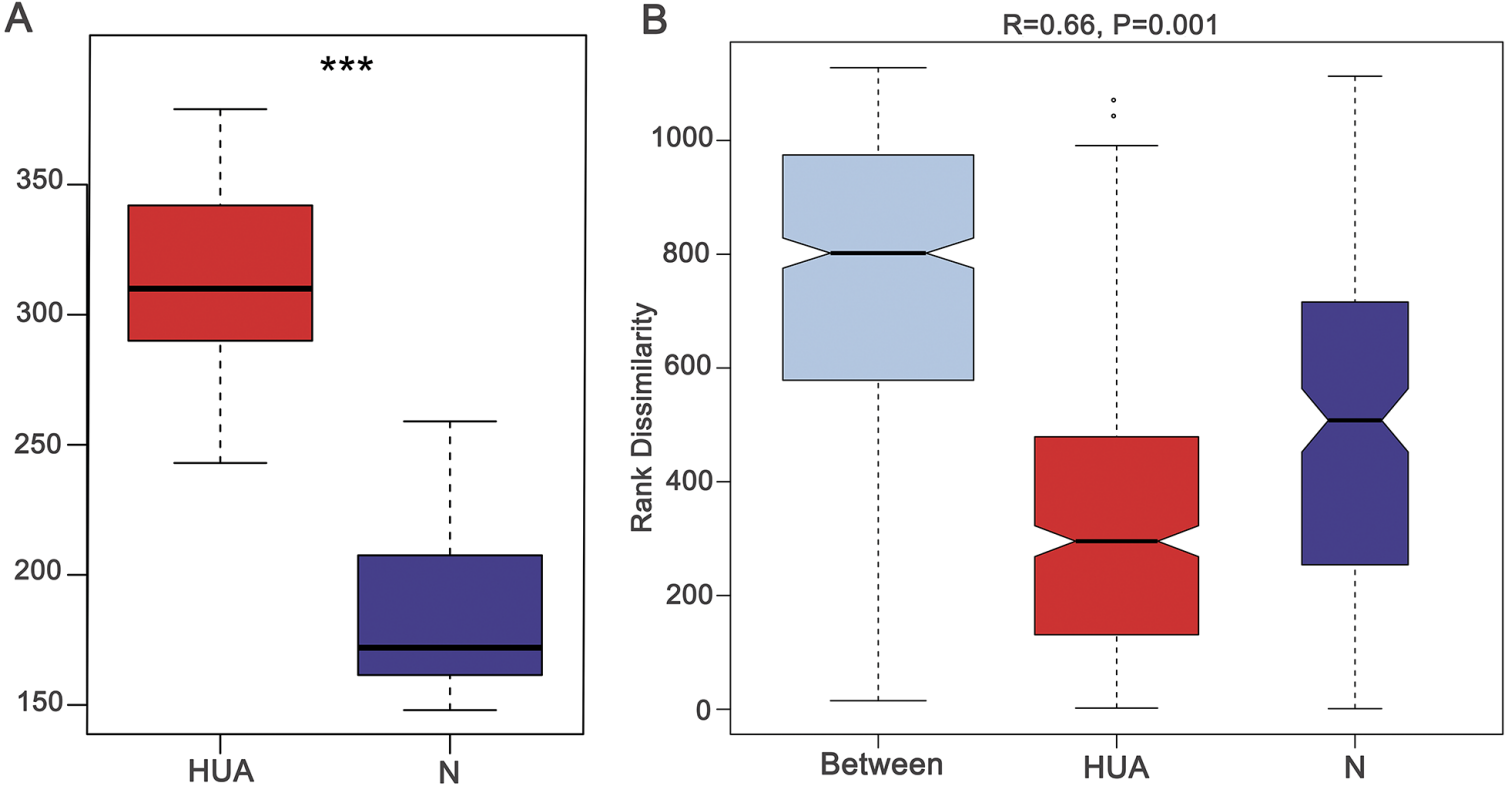
Validating the HUA model and grouping of experimental animals. (A) A boxplot showing uric acid content (*n* = 19 − 29); (B) Anosim analysis based on gut microbiota (*n* = 19 − 29), the *R* value correlation showing intra-group and inter-group differences. HUA, hyperuricemia group; N, normal group; Asterisks show any significant discrepancy by Student’s unpaired *t*-test; ***, *P* < 0.001.

In the fifth week of establishing the HUA model, the uric acid (UA), blood urea nitrogen (BUN), creatinine (Cr) and total cholesterol (TC) were found to be significantly different between the two groups (Table S1). The elevated UA levels in HUA rats indicated that the hyperuricemia model was successfully established (Fig. 1A). BUN and Cr levels in HUA rats were significantly elevated compared to that in N rats, indicating that the renal function indices in HUA rats were abnormal. TC was also significantly elevated in HUA rats, indicating that the lipid metabolism of HUA rats was abnormal.

### The change of gut microbiota in HUA rats

**Figure 2:**
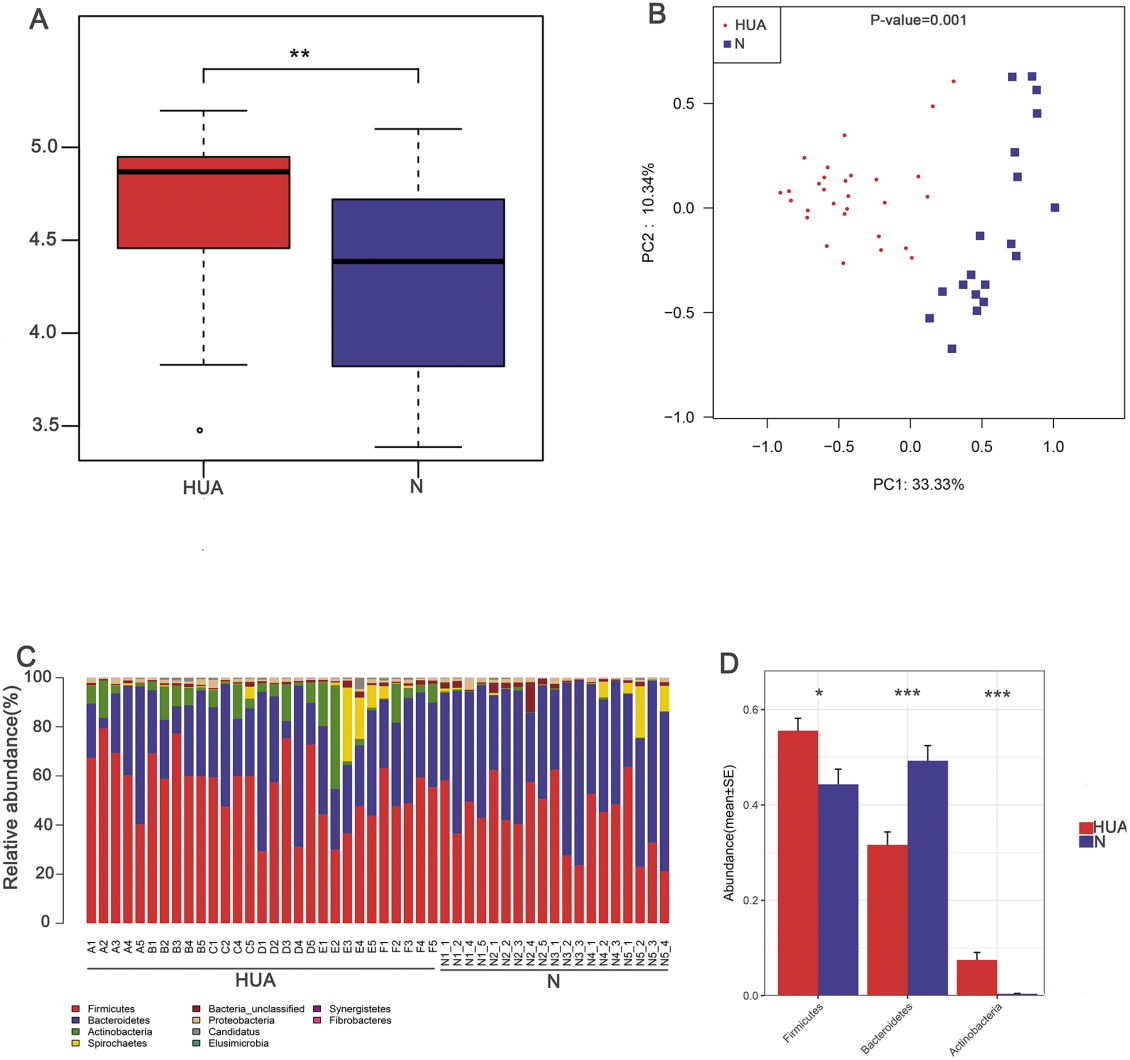
Characteristic comparisons of the gut microbiota between the HUA and the N groups. (A) the Shannon index (*n* = 19 − 29); (B) PCoA analyzing results (*n* = 19 − 29); (C) the microbial composition at phylum level (*n* = 19 − 29); (D) the relative abundance of three dominant phyla between the two groups (*n* = 19 − 29). HUA, hyperuricemia group; N, normal group. Data are represented as mean ± SD. Asterisks indicate the significance of discrepancy by Wilcoxon rank-sum test; ***, *P* < 0.001; **, *P* < 0.01 and *, *P* < 0.05.

In order to assess the consequence of grouping in the HUA model, we compared the microbial differences among and within groups and displayed as a boxplot based on Anosim analysis. We found that intra-group differences were smaller than inter-group differences (Fig. 1B). The higher Shannon index of the HUA group verified an altered microbial diversity in HUA rats (Fig. 2A). PCoA analysis implied a significant microbiota differences between HUA and N rats (Fig. 2B). The relative abundance of Bacteroidetes in the HUA rats was lower than that of N rats, and the relative abundance of Firmicutes and Actinobacteria in the HUA rats was higher than that of N rats (Figs. 2C and 2D). These results suggested that the gut microbiota of the HUA rats had changed in comparison with that of N rats.

**Figure 3:**
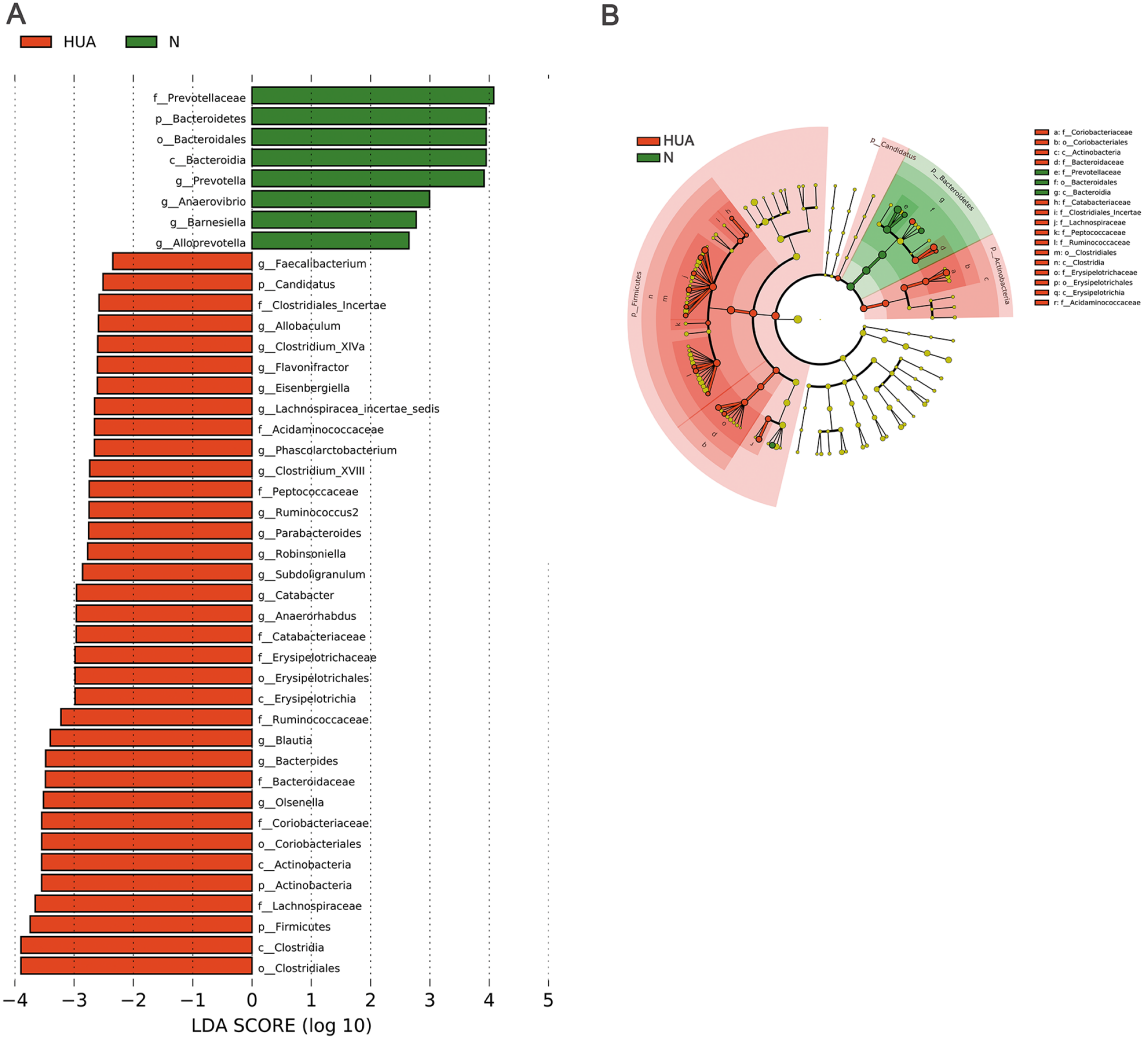
Microbial taxa discrepancies between the HUA and the N rats. (A) Histograms (*n* = 19 − 29); (B) Cladogram (*n* = 19 − 29); HUA, hyperuricemia group; N, normal group.

LEfSe analysis identified the discrepant microbial taxa (LDA score >2) between HUA and N rats. Genera *Prevotella, Anaerovibrio, Alloprevotella*, *Barnesiella* among others were less abundant in the gut of HUA rats than that of N rats. Conversely, genera *Allobaculum, Clostridium_XlVa, Flavonifractor, Phascolarctobacterium, Clostridium_XVIII, Parabacteroides, Robinsoniella, Subdoligranulum, Catabacter, Blautia, Bacteroides, Olsenella* among others were more abundant in the intestinal tract of HUA rats than that of N rats (Fig. 3). Bacteria including *Olsenella*, Clostridiales_unclassified, Peptococcaceae_unclassified, *Blautia* and Lachnospiracea_incertae_sedis were significantly and positively correlated with UA. Only Prevotellaceae_unclassified and *Prevotella* were significantly and negatively correlated with UA (Table S2). These altered microbial taxa and the correlation between gut bacteria and UA suggest that the gut microbiota may have some roles in hyperuricemia.

### Decreased uric acid content in Ab-fed HUA rats

**Figure 4:**
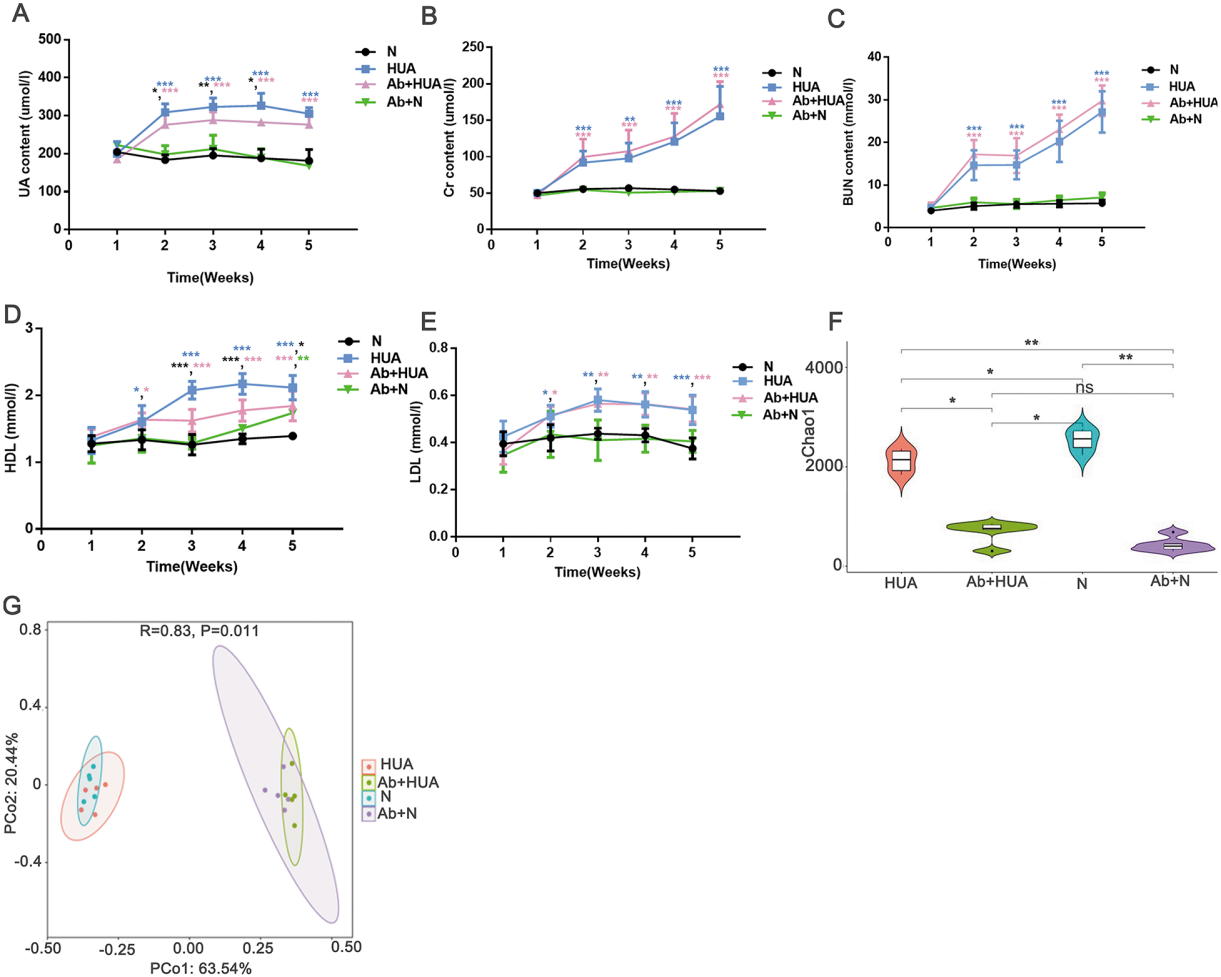
Analysis of biochemical indicators and gut microbiota in Ab-fed HUA rats (*n* = 3 − 8). (A) UA, Uric acid; (B) Cr, Creatinine ; (C) BUN, Blood urea nitrogen; (D) HDL, high density lipoprotein; (E) LDL, low density lipoprotein; (F) Chao1; (G) PCoA analysis; N, normal group; HUA, hyperuricemia group; Ab+HUA, antibiotic-fed hyperuricemia group; Ab+N, antibiotic-fed normal group; dusty blue, pink, green and black asterisks indicate the significance of discrepancy between HUA and N, Ab+HUA and N, Ab+N and N, HUA and Ab+HUA, respectively; Data are represented as mean ± SD. Biochemical data and microbial data were compared using one-way ANOVA and Kruskal-Wallis test, respectively. Statistical significance is defined as *** *P* < 0.001, ** *P* < 0.01, * *P* < 0.05 and ns, no significant difference.

To explore the potential roles played by gut microbiota, we interfered with the gut microbiota of the rats with antibiotics and compared the degree of UA elevation between the HUA rats and the Ab-fed HUA rats. As shown in Fig. 4A, compared with the N rats, UA levels in HUA rats increased significantly from the second week to the fifth week, indicating that the established hyperuricemia model was successfully maintained. We then compared the gut microbiome of the HUA and the N rats and found that the gut microbiome of the HUA rats had changed (Fig. S1). The reproducibility of the result suggested that gut microbiota and hyperuricemia are associated with each other.

The UA level in the HUA rats was found to be significantly elevated than that in N rats. Similarly, the Cr, BUN, high density lipoprotein (HDL) and low-density lipoprotein (LDL) in HUA rats were also significantly elevated than those in N rats. These findings indicated that the renal function indices and lipid metabolism in HUA rats are abnormal (Fig. 4B through Fig. 4E). In contrast, UA level in Ab-fed HUA rats was significantly lower than that in HUA rats; however UA level was still significantly higher in the Ab-fed HUA rats than in N rats (Fig. 4A), and Cr, BUN and LDL was similar between the Ab-fed HUA and the HUA rats (Figs. 4B, 4C and 4E). Furthermore, HDL in the Ab-fed HUA rats was also significantly lower than that in the HUA rats from the third week to the fifth week of the experiment (Fig. 4D).

### The disturbed gut microbiota in Ab-fed HUA rats

We analyzed fecal microbiota from the third week of the experiment. As expected, antibiotics significantly reduced the richness of gut microbiota of the Ab-fed HUA rats (Fig. 4F), which resulted in the difference between the Ab-fed HUA and the HUA rat gut microbiome (Fig. 4G). In combination with the lower uric acid content, the altered gut microbiota of the Ab-fed HUA rats suggested that gut microbiota may have a potential role in hyperuricemia.

### Fecal microbiota from HUA rats increased uric acid content of recipient rats

**Figure 5:**
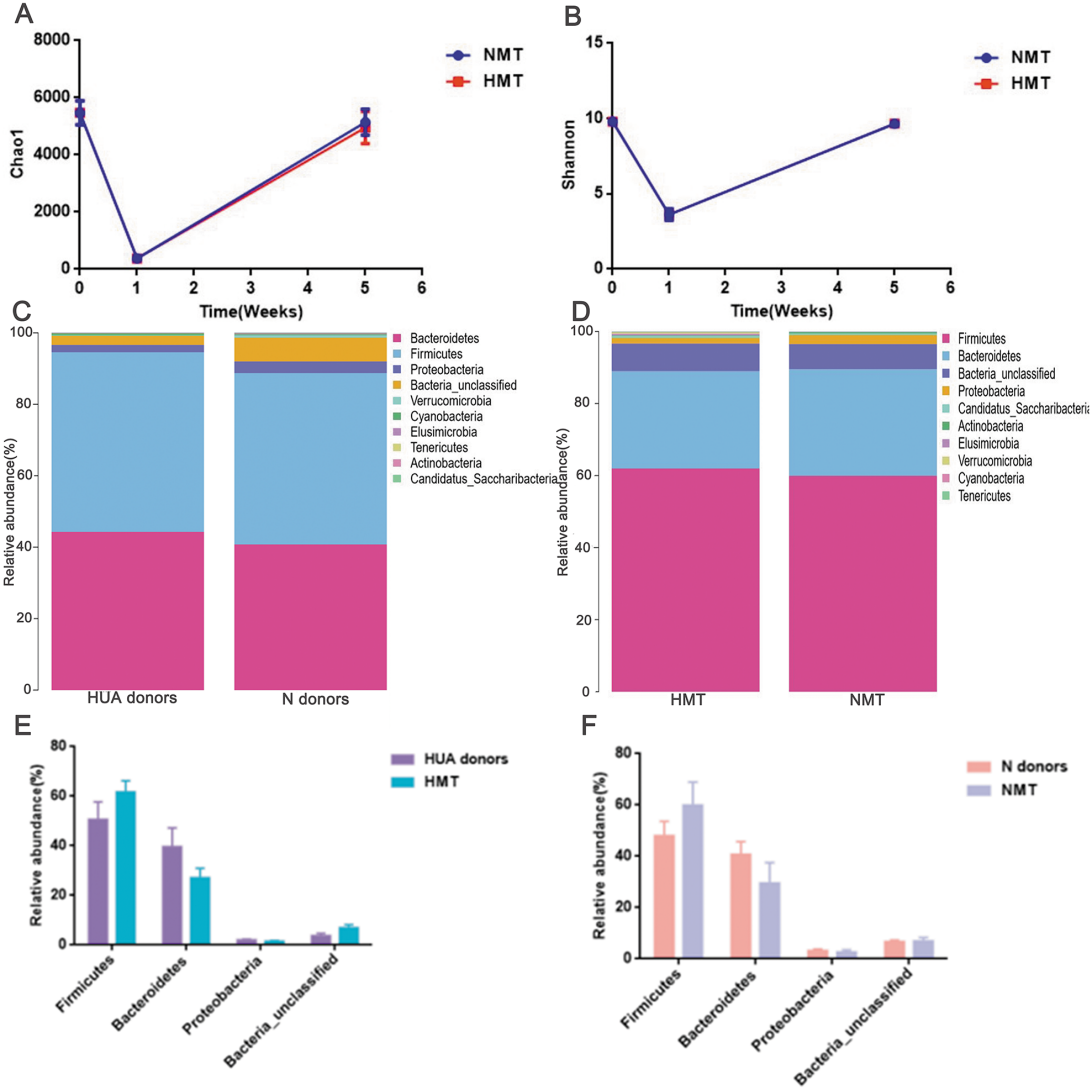
Establishing recipient rat model and similar microbiota between donors and recipient rats. (A) and (B), the richness (Chao1) and diversity (Shannon) of gut microbiota in normal rats (week 0), pre- (week 1) and post-transplant recipient rats (week 5), respectively; (C) (D), the similar dominant phylum between donors and recipient rats; (E) and (F), the similar microbial abundance between donors and recipient rats; HUA and N donors, the fecal microbiota of HUA and N rats, respectively; HMT and NMT, hyperuricemia and normal microbiota transplantation group, respectively.

After antibiotics intervention, the relative abundance of the dominant phyla (Bacteroidetes, Firmicutes, Proteobacteria, Bacteria_unclassified, etc.) and microbial composition of rats in the first week altered significantly (Fig. S2). The dramatic decrease in richness (Fig. 5A) and diversity (Fig. 5B and Fig. S2) of the gut microbiota (*P* < 0.001) in the first week revealed that the recipient rat model was successfully established. After the transplantation, the relative abundance of the dominant phyla (Bacteroidetes, Firmicutes, Proteobacteria, Bacteria_unclassified, etc.) and the microbial composition of the recipient rats also altered significantly (Fig. S2). The richness and diversity of gut microbiota of recipient rats increased to the original (*P* > 0.05) (Figs. 5A and 5B). Besides, a similar dominant phylum existed in the donor (HUA and N) and the recipient rats (HMT and NMT) (Figs. 5C and 5D). The abundance of dominant phyla Firmicutes, Bacteroidetes, Proteobacteria and Bacteria_unclassified in the recipient rats did not differ from that in the donors (*P* > 0.05) (Figs. 5E and 5F), implying that the fecal microbiota from the donors has altered the gut microbiota of the recipient rats, and that the gut microbiome of recipient rats is closer to donors.

**Figure 6:**
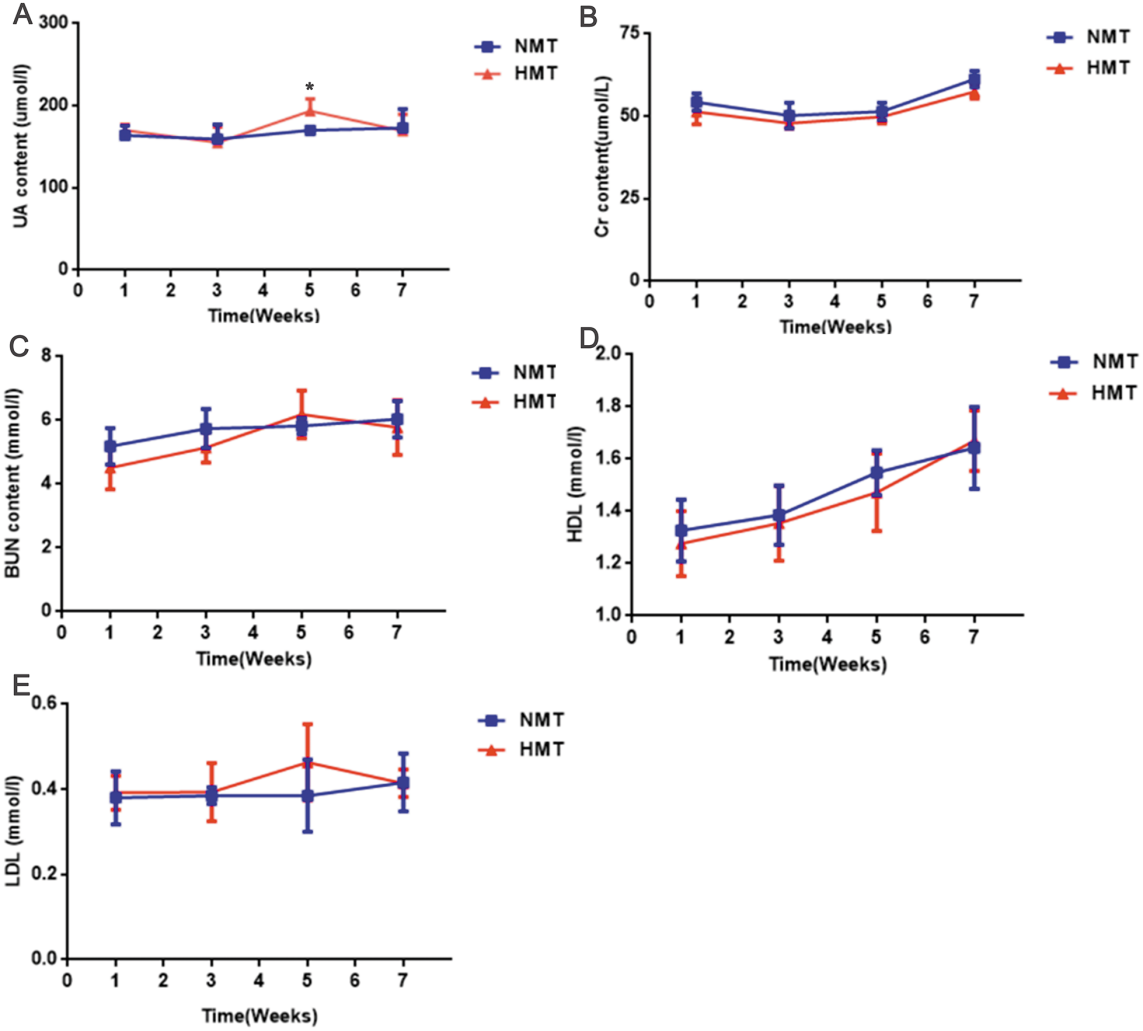
Biochemical analyses of fecal transplant recipient rats (*n* = 3 − 10). Week 1, pre-transplant recipient rats; Week 3, 5 and 7, post-transplant recipient rats; (A), UA, uric acid; (B) Cr, Creatinine; (C) BUN, Blood urea nitrogen; (D) HDL, High density lipoprotein ; (E), LDL, Low density lipoprotein; NMT, normal microbiota transplantation group; HMT, hyperuricemia microbiota transplantation group; data are represented as mean ± SD. Asterisks, the significance of discrepancy between the HMT and the NMT groups by Student’s unpaired *t*-test, *, *P* < 0.05.

The uric acid content of the recipient rats in the HMT group increased significantly in comparison to that in the NMT group at the third week after transplantation (week 5). Cr, BUN, HDL and LDL were not significantly different between the HMT group and the NMT group. Increased uric acid content in the recipient rats indicated that the fecal microbiota from the HUA rats may have been involved in hyperuricemia (Fig. 6).

### The microbial taxa may associate with the occurrence of hyperuricemia

We found that the genera *Vallitalea*, *Christensenella* and *Insolitispirillum* were all enriched in the HUA and the HMT groups (Tables S3 and S4). Such similarity implies that these microbial taxa may associate with the occurrence of hyperuricemia. However, the specific biological function and pathogenesis of these microbial taxa in the occurrence of hyperuricemia needs further study.

## Discussion

Hyperuricemia and gout are common metabolic diseases, which are caused by the disorder of purine metabolism and the decrease of uric acid excretion (Chen et al., 2015). The uric acid salt crystalizes in the joints and the surroundings (Krishnan et al., 2013) in case of prolong presence of serum uric acid levels, and finally, inflammatory arthritis results (Li et al., 2015), when the motion of joints are affected (Bardin & Richette, 2017). Besides, hyperuricemia increases the risk of diverse metabolic diseases including type 2 diabetes, chronic kidney and cardiovascular diseases (Sluijs et al., 2012; Filiopoulos, Hadjiyannakos & Vlassopoulos, 2012; Puddu et al., 2012). Although there are many effective drugs on the market to treat the disease, it is necessary to study the disease further because of the multiple contraindications and harmful effects of these medicines.

A recent study has indicated that the new pathogenesis of hyperuricemia is mainly intestinal microbiota (Pascart & Lioté, 2019), but the specific pathogenesis of gut microbiota is unclear. Some studies have reported altered gut microbiome in gout patients (Shao et al., 2017), with gut bacteria expressing the gene of allantoinase significantly reduced (Crane, 2013), suggesting that altered gut microbiota may associate with gout, and therefore, may help distinguish gout patients from healthy patients and serve as new targets for disease treatment (Guo et al., 2016). As reported previously, certain probiotics containing uricolytic ability can be used to help prevent oxonic acid-induced hyperuricemia in animals (García-Arroyo et al., 2018). Similarly, a potential probiotic has been found that improves fructose-induced hyperuricemia by reducing intestinal inosine (Wang et al., 2019). However, it is still unclear whether gut microbiota plays a role in the occurrence of hyperuricemia.

In this study, we found that the gut microbiota of the HUA rats changed drastically, which indicated that the gut microbiota may be associated with hyperuricemia. In addition, we also found that the uric acid content in the Ab-fed HUA rats was lower than that in the HUA rats, indicating that the gut microbiota plays a potential role in hyperuricemia. By transplanting the fecal microbiota of HUA rats into the recipient rats, we found that fecal microbiota from the HUA rats caused an increase in the uric acid content of recipient rats. This finding confirmed that the gut microbiota plays a potential role in hyperuricemia, and may be involved in the occurrence of hyperuricemia.

Xi et al. (2019) reported that skewing of gram-negative gut-bacteria, the up-regulation of Proteobacteria, is the signature of gout, whereby the pathogenesis is due to adverse effect of lipopolysaccharide (LPS) on kidney function. Moreover, infection with *E. coli* and *Shiga-toxigenic E. coli* that belong to the gram-negative Proteobacteria phylum increases serum uric acid content, implying that the phylum Proteobacteria is associated with hyperuricemia. However, the mechanism of elevated uric acid content is uncertain (Crane et al., 2013). Similarly, recent studies have shown that high content of serum LPS is strongly associated with kidney disease (Lassenius et al., 2011), and that intestinal LPS translocation may be involved in gout by interfering with the kidney function (Xi et al., 2019), suggesting that the gram-negative bacteria in the gut may affect gout through LPS. In our study, we tried to find the bacteria that may be associated with hyperuricemia. We showed that genera *Vallitalea, Christensenella* and *Insolitispirillum* were all enriched in the HUA and the HMT group with high uric acid content. Coincidentally, these bacteria are also gram-negative (Lakhal et al., 2013; Morotomi, Nagai & Watanabe, 2012; Yoon et al., 2007), and furthermore, the genus *Insolitispirillum* belongs to the phylum Proteobacteria, indicating that genera *Vallitalea, Christensenella* and *Insolitispirillum* may be associated with hyperuricemia. Furthermore, *Christensenella* is highly heritable, and relates to high serum glucose content (Goodrich et al., 2014; Crusell et al., 2018), consistent with previous report showing high genetic trait of hyperuricemia (Krishnan et al., 2012). On the other hand, the genera *Vallitalea* and *Insolitispirillum* have been rarely reported in some diseases. Together, our finding suggest that gut microbiota, including genera *Vallitalea, Christensenella* and *Insolitispirillum*, are related to hyperuricemia*.*

## Conclusions

Based on our findings, the diversity and abundance of gut microbiota was altered in HUA rats, suggesting that gut microbiota may have a potential role in hyperuricemia. Furthermore, many microbial taxa, such as those in genera *Vallitalea*, *Christensenella* and *Insolitispirillum* could be associated with hyperuricemia. We propose isolation and culture of individual bacteria to further explore the function and mechanism of intestinal bacteria in HUA pathogenesis.

##  Supplemental Information

10.7717/peerj.8664/supp-1 Figure S1 The altered gut microbiome of HUA rats (A) The microbial composition at phylum level (*n* = 5). (B) The relative abundance of altered phyla in the two groups (*n* = 5); HUA, hyperuricemia group; N, Normal group; Asterisks, the significance of discrepancy by Wilcoxon rank-sum test; *, *P* < 0.05

10.7717/peerj.8664/supp-2 Figure S2 Changes in the relative abundance of dominant phyla and the microbial composition of rats before and after the intervention of antibiotics and after the transplantation (*n* = 5) W0 (week 0), before the intervention with antibiotics; W1 (week1), after the intervention with antibiotics; W5-NMT and W5-HMT, post-transplant recipient rats; 1,2,3,4,5 indicate the significance of discrepancy (*P* < 0.05) between W0 and W1, W1 and W5-NMT, W1 and W5-HMT, W0 and W5-NMT, W0 and W5-HMT by Kruskal-Wallis test, respectively;

10.7717/peerj.8664/supp-3 Table S1 Serum biochemical indices of rats at the fifth week (*n* = 19 − 29) HUA, hyperuricemia group; N, normal group; The data is presented as the mean ± standard deviation; Asterisks, the significance of discrepancy by Student’s unpaired t-test; *, *P* < 0.05; ns, no significant difference; UA, uric acid; BUN, blood urea nitrogen; Cr , creatinine ; TG, triglyceride; TC, total cholesterol.

10.7717/peerj.8664/supp-4 Table S2 The correlation between bacterial abundance and uric acid content (*n* = 19 − 29) Asterisks, the significance of discrepancy by Wilcoxon rank-sum test; ***, *P* < 0.001

10.7717/peerj.8664/supp-5 Table S3 Microbial taxa enriched in the HUA group identified by Lefse analysis (*n* = 5) N, normal group; HUA, hyperuricemia group; Ab+HUA, antibiotic-fed hyperuricemia group; Ab+N, antibiotic-fed normal group; Highlighted in bold, the microbial taxa that were enriched in both HUA and HMT groups; The statistical analysis method is Kruskal-Wallis test.

10.7717/peerj.8664/supp-6 Table S4 Microbial taxa enriched in the HMT group identified by Lefse analysis (*n* = 5) NMT, normal microbiota transplantation group; HMT, hyperuricemia microbiota transplantation group; Highlighted in bold, the microbial taxa that were enriched in both HUA and HMT groups; The statistical analysis method is Wilcoxon rank-sum test.

10.7717/peerj.8664/supp-7 Data S1 The raw uric acid data from HUA rats N, normal group; HUA, hyperuricemia group; UA, uric acid;

10.7717/peerj.8664/supp-8 Data S2 The raw biochemical data from Ab-fed HUA rats N, normal group; HUA, hyperuricemia group; Ab+HUA, antibiotic-fed hyperuricemia group; Ab+N, antibiotic-fed normal group; UA, uric acid; Cr, creatinine; BUN, blood urea nitrogen; HDL, high density lipoprotein; LDL, low density lipoprotein;

10.7717/peerj.8664/supp-9 Data S3 The raw biochemical data from recipient rats HMT and NMT,hyperuricemia and normal microbiota transplantation group, respectively. UA, uric acid; Cr, creatinine; BUN, blood urea nitrogen; HDL, high density lipoprotein; LDL, low density lipoprotein;

## Additional Information and Declarations

### Data Availability

The following information was supplied regarding data availability: The raw sequence data is available at NCBI SRA: BioProject ID PRJNA589722 and BioProject ID PRJNA579794.
