# Supplementary material for: The altered gut microbiota of high-purine-induced hyperuricemia rats and its correlation with hyperuricemia
Source: PeerJ. 2020 Mar 6;8:e8664. doi: 10.7717/peerj.8664 (PMC7061907; doi:10.7717/peerj.8664)
Supplement: Figure S1 — (A) The microbial composition at phylum level (n = 5). (B) The relative abundance of altered phyla in the two groups (n = 5); HUA, hyperuricemia group; N, Normal group; Asterisks, the significance of discrepancy by Wilcoxon rank-sum test; *, P < 0.05 [file peerj-08-8664-s001.doc]

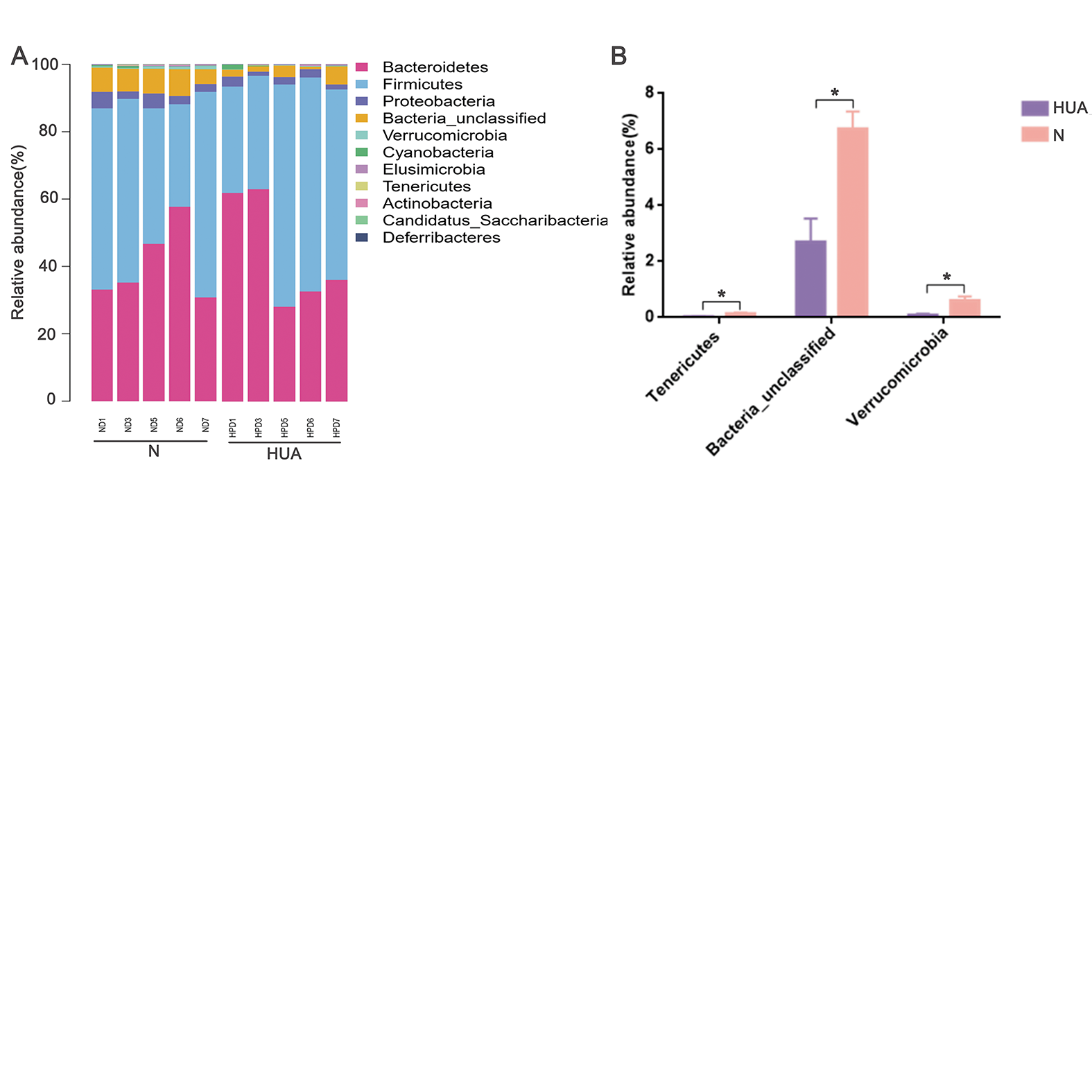


**Supplemental Figure S1** **The altered gut microbiome of HUA rats.**

(A) The microbial composition at phylum level (*n*=5). (B) The relative abundance of altered phyla in the two groups (*n*=5); HUA= hyperuricemia group, N= Normal group; Asterisks, the significance of discrepancy by Wilcoxon rank-sum test; *, *P*<0.05
