# Supplementary material for: The altered gut microbiota of high-purine-induced hyperuricemia rats and its correlation with hyperuricemia
Source: PeerJ. 2020 Mar 6;8:e8664. doi: 10.7717/peerj.8664 (PMC7061907; doi:10.7717/peerj.8664)
Supplement: Figure S2 — W0 (week 0), before the intervention with antibiotics; W1 (week1), after the intervention with antibiotics; W5-NMT and W5-HMT, post-transplant recipient rats; 1,2,3,4,5 indicate the significance of discrepancy (P < 0.05) between W0 and W1, W1 and W5-NMT, W1 and W5-HMT, W0 and W5-NMT, W0 and W5-HMT by Kruskal-Wallis test, respectively; [file peerj-08-8664-s002.doc]

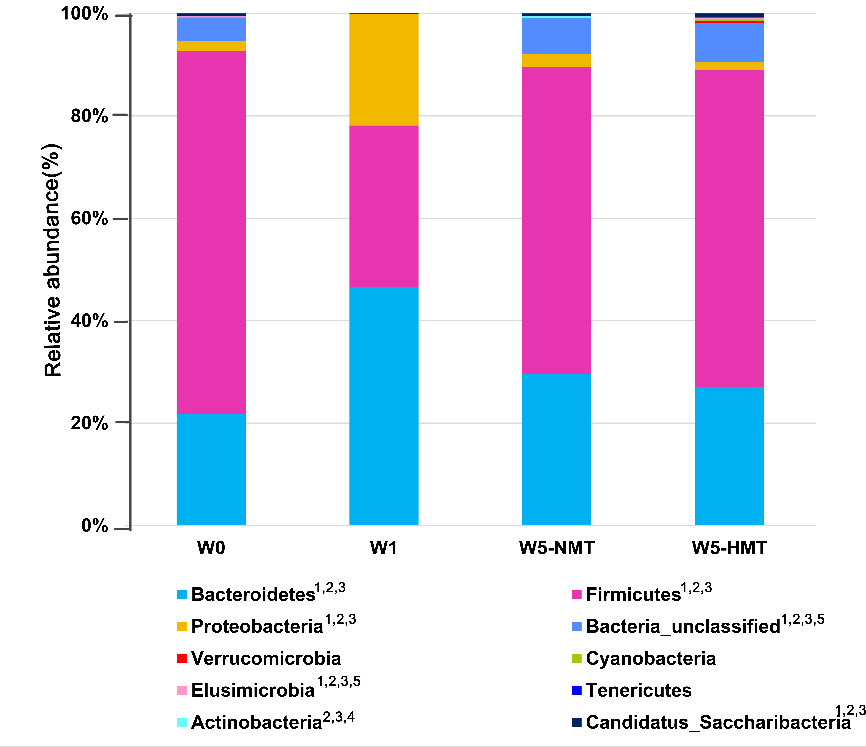


**Supplemental Figure S2 Changes in the relative abundance of dominant phyla and the microbial composition of rats before and after the intervention of antibiotics and after the transplantation (n=5)**

W0 (week 0), before the intervention with antibiotics; W1 (week1), after the intervention with antibiotics; W5-NMT and W5-HMT, post-transplant recipient rats; 1,2,3,4,5 indicate the signiﬁcance of discrepancy (*P*<0.05) between W0 and W1, W1 and W5-NMT, W1 and W5-HMT, W0 and W5-NMT, W0 and W5-HMT by Kruskal-Wallis test, respectively;
