# Supplementary material for: The altered gut microbiota of high-purine-induced hyperuricemia rats and its correlation with hyperuricemia
Source: PeerJ. 2020 Mar 6;8:e8664. doi: 10.7717/peerj.8664 (PMC7061907; doi:10.7717/peerj.8664)
Supplement: Table S1 — HUA, hyperuricemia group; N, normal group; The data is presented as the mean ± standard deviation; Asterisks, the significance of discrepancy by Student’s unpaired t-test; *, P < 0.05; ns, no significant difference; UA, uric acid; BUN, blood urea nitrogen; Cr , creatinine ; TG, triglyceride; TC, total cholesterol. [file peerj-08-8664-s003.doc]

**Supplemental Table S1 Serum biochemical indices of rats at the fifth week (*n*=19-29)**

| Index | HUA | N | Significance level |
| --- | --- | --- | --- |
| UA (µmol·L) | 310.03±36.86 | 187.32±35.06 | * |
| BUN (mmol·L) | 16.25±5.28 | 6.17±0.76 | * |
| Cr (µmol·L) | 107.78±11.91 | 27.37±5.34 | * |
| TG (mmol·L) | 0.82±0.25 | 0.68±0.30 | ns |
| TC (mmol·L) | 1.71±0.21 | 1.54±0.17 | * |

HUA, hyperuricemia group; N, normal group; The data is presented as the mean ± standard deviation; Asterisks, the significance of discrepancy by Student’s unpaired t-test; *, *P*<0.05; ns, no significant difference; UA, uric acid; BUN, blood urea nitrogen; Cr, creatinine; TG, triglyceride; TC, total cholesterol.
