# Supplementary material for: The altered gut microbiota of high-purine-induced hyperuricemia rats and its correlation with hyperuricemia
Source: PeerJ. 2020 Mar 6;8:e8664. doi: 10.7717/peerj.8664 (PMC7061907; doi:10.7717/peerj.8664)
Supplement: Table S2 — Asterisks, the significance of discrepancy by Wilcoxon rank-sum test; ***, P < 0.001 [file peerj-08-8664-s004.doc]

# Supplemental Table S2 The correlation between bacterial abundance and uric acid content (*n*=19-29)

| Gut bacteria | *R* value | Significance level |
| --- | --- | --- |
| Olsenella | 0.68 | *** |
| Clostridiales_unclassified | 0.66 | *** |
| Peptococcaceae_unclassified | 0.63 | *** |
| Blautia | 0.59 | *** |
| Lachnospiracea_incertae_sedis | 0.56 | *** |
| Prevotellaceae_unclassified | -0.53 | *** |
| Prevotella | -0.55 | *** |

Asterisks, the significance of discrepancy by Wilcoxon rank-sum test; ***, *P*<0.001
