# Supplementary material for: The altered gut microbiota of high-purine-induced hyperuricemia rats and its correlation with hyperuricemia
Source: PeerJ. 2020 Mar 6;8:e8664. doi: 10.7717/peerj.8664 (PMC7061907; doi:10.7717/peerj.8664)
Supplement: Table S3 — N, normal group; HUA, hyperuricemia group; Ab+HUA, antibiotic-fed hyperuricemia group; Ab+N, antibiotic-fed normal group; Highlighted in bold, the microbial taxa that were enriched in both HUA and HMT groups; The statistical analysis method is Kruskal-Wallis test. [file peerj-08-8664-s005.doc]

**Supplemental Table S3 Microbial taxa enriched in the HUA group identified by Lefse analysis (*n*=5)**

| **Discrepant microbial taxa** | **Enriched** | **LDA**  **score** | ***P* values** |
| --- | --- | --- | --- |
| ***g__Vallitalea*** | **HUA** | **3.11** | **0.002** |
| ***g__Christensenella*** | **HUA** | **2.74** | **0.001** |
| ***g__Insolitispirillum*** | **HUA** | **2.60** | **0.002** |
| *f__Acidaminococcaceae* | HUA | 3.71 | 0.001 |
| *g__Acetatifactor* | HUA | 3.51 | 0.001 |
| *c__Deltaproteobacteria* | HUA | 3.73 | 0.001 |
| *g__Neglecta* | HUA | 2.56 | 0.001 |
| *g__Rothia* | HUA | 2.87 | 0.010 |
| *o__Rhodospirillales* | HUA | 2.73 | 0.001 |
| *g__Stomatobaculum* | HUA | 2.58 | 0.001 |
| *f__Eubacteriaceae* | HUA | 3.51 | 0.001 |
| *g__Holdemania* | HUA | 2.64 | 0.001 |
| *g__Murimonas* | HUA | 2.53 | 0.006 |
| *p__Actinobacteria* | HUA | 2.93 | 0.003 |
| *f__Pasteurellaceae* | HUA | 2.88 | 0.013 |
| *o__Pasteurellales* | HUA | 2.89 | 0.013 |
| *g__Provencibacterium* | HUA | 3.10 | 0.011 |
| *f__Oscillospiraceae* | HUA | 2.93 | 0.001 |
| *f__Tannerellaceae* | HUA | 3.02 | 0.035 |
| *g__Bilophila* | HUA | 3.40 | 0.001 |
| *g__Roseburia* | HUA | 3.90 | 0.001 |
| *f__Barnesiellaceae* | HUA | 3.62 | 0.001 |
| *o__Clostridiales* | HUA | 5.17 | 0.004 |
| *c__Cytophagia* | HUA | 3.36 | 0.002 |
| *g__Anaerobium* | HUA | 3.04 | 0.006 |
| *g__Oscillospira* | HUA | 3.71 | 0.022 |
| *c__Actinobacteria* | HUA | 2.93 | 0.003 |
| *g__Ruminococcus* | HUA | 4.18 | 0.001 |
| *c__Negativicutes* | HUA | 3.72 | 0.003 |
| *g__Emticicia* | HUA | 3.36 | 0.002 |
| *g__Clostridium_XVIII* | HUA | 3.32 | 0.011 |
| *g__Desulfovibrio* | HUA | 3.14 | 0.001 |
| *g__Coprococcus* | HUA | 3.13 | 0.001 |
| *f__Corynebacteriaceae* | HUA | 3.03 | 0.015 |
| *g__Robinsoniella* | HUA | 2.82 | 0.001 |
| *f__Christensenellaceae* | HUA | 2.74 | 0.001 |
| *g__Acutalibacter* | HUA | 2.62 | 0.010 |
| *g__Lachnoclostridium* | HUA | 3.91 | 0.001 |
| *g__Eisenbergiella* | HUA | 2.73 | 0.014 |
| *g__Clostridium_XlVb* | HUA | 2.98 | 0.001 |
| *o__Desulfovibrionales* | HUA | 3.60 | 0.001 |
| *g__Butyricicoccus* | HUA | 2.76 | 0.001 |
| *f__Ruminococcaceae* | HUA | 5.01 | 0.001 |
| *f__Moraxellaceae* | HUA | 3.22 | 0.034 |
| *o__Selenomonadales* | HUA | 3.72 | 0.003 |
| *g__Corynebacterium* | HUA | 3.02 | 0.015 |
| *f__Rhodospirillaceae* | HUA | 2.73 | 0.001 |
| *g__Oscillibacter* | HUA | 2.93 | 0.001 |
| *o__Cytophagales* | HUA | 3.36 | 0.002 |
| *g__Dongia* | HUA | 2.69 | 0.005 |
| *g__Muricomes* | HUA | 2.58 | 0.001 |
| *g__Moryella* | HUA | 2.71 | 0.002 |
| *g__Oscillibacter* | HUA | 4.21 | 0.001 |
| *g__Faecalibacterium* | HUA | 2.80 | 0.001 |
| *g__Rodentibacter* | HUA | 2.88 | 0.013 |
| *p__Firmicutes* | HUA | 5.10 | 0.006 |
| *o__Actinomycetales* | HUA | 2.93 | 0.007 |
| *g__Parabacteroides* | HUA | 3.00 | 0.035 |
| *g__Barnesiella* | HUA | 3.95 | 0.001 |
| *g__Eubacterium* | HUA | 3.51 | 0.001 |
| *f__Desulfovibrionaceae* | HUA | 3.59 | 0.001 |
| *g__Saccharofermentans* | HUA | 2.99 | 0.026 |
| *c__Clostridia* | HUA | 5.17 | 0.004 |
| *f__Porphyromonadaceae* | HUA | 4.81 | 0.018 |
| *g__Anaerotruncus* | HUA | 3.08 | 0.001 |
| *g__Intestinimonas* | HUA | 3.41 | 0.001 |
| *g__Barnesiella* | HUA | 3.62 | 0.001 |
| *f__Micrococcaceae* | HUA | 2.88 | 0.010 |
| *g__Phascolarctobacterium* | HUA | 3.71 | 0.001 |
| *g__Harryflintia* | HUA | 2.68 | 0.001 |
| *f__Cytophagaceae* | HUA | 3.36 | 0.002 |
| *o__Bacillales* | N | 3.01 | 0.013 |
| *g__Turicibacter* | N | 2.74 | 0.001 |
| *c__Zetaproteobacteria* | N | 2.99 | 0.001 |
| *f__Clostridiaceae* | N | 3.73 | 0.006 |
| *f__Elusimicrobiaceae* | N | 3.03 | 0.001 |
| *g__Peptococcus* | N | 2.78 | 0.001 |
| *g__Dehalobacterium* | N | 3.01 | 0.035 |
| *g__Ethanoligenens* | N | 2.69 | 0.001 |
| *g__Schwartzia* | N | 3.16 | 0.006 |
| *g__Elusimicrobium* | N | 3.03 | 0.001 |
| *c__Bacilli* | N | 4.48 | 0.028 |
| *g__Helicobacter* | N | 3.16 | 0.001 |
| *g__Acetanaerobacterium* | N | 2.89 | 0.001 |
| *g__Streptococcus* | N | 2.60 | 0.015 |
| *c__Alphaproteobacteria* | N | 2.92 | 0.001 |
| *g__Anaerorhabdus* | N | 2.76 | 0.001 |
| *g__Papillibacter* | N | 2.93 | 0.003 |
| *p__Elusimicrobia* | N | 3.03 | 0.001 |
| *f__Peptococcaceae* | N | 2.86 | 0.001 |
| *o__Campylobacterales* | N | 3.16 | 0.001 |
| *g__Alloprevotella* | N | 4.46 | 0.001 |
| *g__Acetivibrio* | N | 4.43 | 0.001 |
| *g__Faecalicatena* | N | 3.22 | 0.006 |
| *o__Coriobacteriales* | N | 2.86 | 0.001 |
| *g__Clostridium* | N | 3.78 | 0.001 |
| *g__Acetitomaculum* | N | 2.86 | 0.037 |
| *g__Bacillus* | N | 3.01 | 0.013 |
| *o__Mariprofundales* | N | 2.99 | 0.001 |
| *f__Mariprofundaceae* | N | 2.99 | 0.001 |
| *g__Lachnotalea* | N | 2.80 | 0.001 |
| *g__Anaerovorax* | N | 3.00 | 0.001 |
| *g__Anaerostipes* | N | 3.09 | 0.002 |
| *g__Alistipes* | N | 3.58 | 0.005 |
| *g__Collinsella* | N | 3.35 | 0.006 |
| *g__Anaerotaenia* | N | 3.02 | 0.001 |
| *f__Bacillaceae* | N | 3.02 | 0.013 |
| *f__Streptococcaceae* | N | 2.54 | 0.005 |
| *c__Epsilonproteobacteria* | N | 3.16 | 0.001 |
| *f__Rikenellaceae* | N | 3.59 | 0.004 |
| *g__Peptococcus* | N | 2.95 | 0.001 |
| *g__Mariprofundus* | N | 2.99 | 0.001 |
| *g__Sporobacter* | N | 2.70 | 0.001 |
| *g__Pseudoflavonifractor* | N | 2.82 | 0.001 |
| *g__Anaerosporobacter* | N | 2.85 | 0.001 |
| *g__Butyrivibrio* | N | 2.82 | 0.001 |
| *g__Prevotella* | N | 4.69 | 0.001 |
| *o__Elusimicrobiales* | N | 3.03 | 0.001 |
| *f__Peptococcaceae_1* | N | 2.95 | 0.001 |
| *g__Lactobacillus* | N | 4.49 | 0.001 |
| *f__Prevotellaceae* | N | 4.90 | 0.001 |
| *g__Frisingicoccus* | N | 2.97 | 0.027 |
| *g__Lachnospira* | N | 2.73 | 0.001 |
| *g__Butyricimonas* | N | 2.92 | 0.001 |
| *g__Syntrophococcus* | N | 2.75 | 0.001 |
| *f__Helicobacteraceae* | N | 3.16 | 0.001 |
| *f__Coriobacteriaceae* | N | 2.86 | 0.001 |
| *c__Elusimicrobia* | N | 3.03 | 0.001 |
| *o__Lactobacillales* | N | 4.48 | 0.028 |
| *f__Lactobacillaceae* | N | 4.49 | 0.001 |
| *f__Bacteroidaceae* | Ab+HUA | 5.28 | 0.006 |
| *o__Erysipelotrichales* | Ab+HUA | 4.45 | 0.006 |
| *g__Oligella* | Ab+HUA | 3.02 | 0.004 |
| *g__Paenalcaligenes* | Ab+HUA | 3.31 | 0.009 |
| *g__Ruminiclostridium* | Ab+HUA | 3.65 | 0.002 |
| *g__Sellimonas* | Ab+HUA | 3.30 | 0.001 |
| *g__Bacteroides* | Ab+HUA | 5.28 | 0.006 |
| *g__Citrobacter* | Ab+HUA | 3.81 | 0.001 |
| *g__Ruminococcus2* | Ab+HUA | 3.26 | 0.011 |
| *f__Alcaligenaceae* | Ab+HUA | 3.07 | 0.003 |
| *g__Parabacteroides* | Ab+HUA | 4.71 | 0.006 |
| *g__Flavonifractor* | Ab+HUA | 3.90 | 0.013 |
| *f__Erysipelotrichaceae* | Ab+HUA | 4.45 | 0.006 |
| *g__Thermotalea* | Ab+HUA | 3.43 | 0.001 |
| *o__Enterobacteriales* | Ab+ N | 5.13 | 0.001 |
| *g__Akkermansia* | Ab+ N | 4.25 | 0.010 |
| *g__Klebsiella* | Ab+ N | 4.57 | 0.001 |
| *p__Proteobacteria* | Ab+ N | 5.11 | 0.002 |
| *o__Chloroplast* | Ab+ N | 3.65 | 0.033 |
| *c__Verrucomicrobiae* | Ab+ N | 4.25 | 0.010 |
| *g__Escherichia* | Ab+ N | 4.84 | 0.001 |
| *g__Streptophyta* | Ab+ N | 3.65 | 0.033 |
| *p__Verrucomicrobia* | Ab+ N | 4.25 | 0.010 |
| *g__Alkaliphilus* | Ab+ N | 3.19 | 0.038 |
| *f__Chloroplast* | Ab+ N | 3.65 | 0.033 |
| *g__Enterococcus* | Ab+ N | 4.07 | 0.001 |
| *c__Chloroplast* | Ab+ N | 3.68 | 0.033 |
| *f__Verrucomicrobiaceae* | Ab+ N | 4.25 | 0.010 |
| *f__Enterobacteriaceae* | Ab+ N | 5.13 | 0.001 |
| *o__Verrucomicrobiales* | Ab+ N | 4.25 | 0.010 |
| *g__Clostridium_XlVa* | Ab+ N | 3.05 | 0.010 |
| *g__Subdoligranulum* | Ab+ N | 4.21 | 0.007 |
| *f__Enterococcaceae* | Ab+ N | 4.07 | 0.001 |
| *c__Gammaproteobacteria* | Ab+ N | 5.13 | 0.002 |
| *g__Proteus* | Ab+ N | 3.54 | 0.001 |

N, normal group; HUA, hyperuricemia group; Ab+HUA, antibiotic-fed hyperuricemia group; Ab+N, antibiotic-fed normal group; Highlighted in bold, the microbial taxa that were enriched in both HUA and HMT groups; The statistical analysis method is Kruskal-Wallis test.
