# Supplementary material for: The altered gut microbiota of high-purine-induced hyperuricemia rats and its correlation with hyperuricemia
Source: PeerJ. 2020 Mar 6;8:e8664. doi: 10.7717/peerj.8664 (PMC7061907; doi:10.7717/peerj.8664)
Supplement: Table S4 — NMT, normal microbiota transplantation group; HMT, hyperuricemia microbiota transplantation group; Highlighted in bold, the microbial taxa that were enriched in both HUA and HMT groups; The statistical analysis method is Wilcoxon rank-sum test. [file peerj-08-8664-s006.doc]

**Supplemental Table S4 Microbial taxa enriched in the HMT group identified by Lefse analysis (*n*=5)**

| **Discrepant**  **microbial taxa** | **Enriched** | **LDA**  **score** | ***P* values** |
| --- | --- | --- | --- |
| ***g__Vallitalea*** | **HMT** | **2.22** | **0.029** |
| ***g__Christensenella*** | **HMT** | **2.37** | **0.049** |
| ***g__Insolitispirillum*** | **HMT** | **2.47** | **0.014** |
| *f__Alteromonadaceae* | HMT | 2.22 | 0.029 |
| *g__Alteromonas* | HMT | 2.18 | 0.029 |
| *o__Alteromonadales* | HMT | 2.26 | 0.029 |
| *g__Bifidobacterium* | NMT | 3.01 | 0.027 |
| *f__Bifidobacteriaceae* | NMT | 3.01 | 0.027 |
| *o__Bifidobacteriales* | NMT | 3.01 | 0.027 |
| *c__Actinobacteria* | NMT | 2.98 | 0.027 |
| *g__Romboutsia* | NMT | 3.67 | 0.027 |
| *g__Clostridium_XlVb* | NMT | 2.97 | 0.027 |
| *f__Clostridiaceae_1* | NMT | 3.29 | 0.014 |
| *p__Actinobacteria* | NMT | 2.96 | 0.027 |
| *f__Peptostreptococcaceae* | NMT | 3.69 | 0.027 |
| *g__Moryella* | NMT | 2.23 | 0.027 |

NMT, normal microbiota transplantation group; HMT, hyperuricemia microbiota transplantation group; Highlighted in bold, the microbial taxa that were enriched in both HUA and HMT groups; The statistical analysis method is Wilcoxon rank-sum test.
